# Physiologically-based pharmacokinetic modelling and dosing evaluation of gentamicin in neonates using PhysPK

**DOI:** 10.3389/fphar.2022.977372

**Published:** 2022-09-28

**Authors:** Hinojal Zazo, Eduardo Lagarejos, Manuel Prado-Velasco, Sergio Sánchez-Herrero, Jenifer Serna, Almudena Rueda-Ferreiro, Ana Martín-Suárez, M. Victoria Calvo, Jonás Samuel Pérez-Blanco, José M. Lanao

**Affiliations:** ^1^ Pharmaceutical Sciences Department, University of Salamanca, Salamanca, Spain; ^2^ Institute of Biomedical Research of Salamanca (IBSAL), Salamanca, Spain; ^3^ Multiscale Modelling in Bioengineering Research Group and Department of Graphic Engineering, University of Seville, Seville, Spain; ^4^ Simulation Department, Empresarios Agrupados Internacional S.A., Madrid, Spain

**Keywords:** PBPK, gentamicin, neonates, PhysPK software, dosing evaluation, TDM

## Abstract

Each year, infections caused around the 25% of neonatal deaths. Early empirical treatments help to reduce this mortality, although optimized dosing regimens are still lacking. The aims were to develop and validate a gentamicin physiologically-based pharmacokinetic (PBPK) model and then potentially explore dosing regimens in neonates using pharmacokinetic and pharmacodynamic criteria. The PBPK model developed consisted of 2 flow-limited tissues: kidney and other tissues. It has been implemented on a new tool called PhysPK, which allows structure reusability and evolution as predictive engine in Model-Informed Precision Dosing (MIPD). Retrospective pharmacokinetic information based on serum levels data from 47 neonates with gestational age between 32 and 39 weeks and younger than one-week postnatal age were used for model validation. The minimal PBPK model developed adequately described the gentamicin serum concentration-time profile with an average fold error nearly 1. Extended interval gentamicin dosing regimens (6 mg/kg q36h and 6 mg/kg q48h for term and preterm neonates, respectively) showed efficacy higher than 99% with toxicity lower than 10% through Monte Carlo simulation evaluations. The gentamicin minimal PBPK model developed in PhysPK from literature information, and validated in preterm and term neonates, presents adequate predictive performance and could be useful for MIPD strategies in neonates.

## 1 Introduction

Neonatal population is defined as newborns with less than 1 month of life or postnatal age (PNA). Despite of neonatal mortality has been effectively reduced through early interventions, globally 2.4 million of neonates died in 2019. The 75% of neonatal mortality occurred during the first week of life and around of the 25% of these deaths, both in resource-rich and resource-poor settings, is caused by infections like sepsis, pneumonia or meningitis (Chan et al., 2013; Author Anonymous, 2020). However, there are few information about the appropriate dosing regimens use in the clinical practice for neonates (Lanao et al., 2004). Different physiological maturation, related to the gestational age (GA), and rapid developmental changes of physiological factors affect drug pharmacokinetics (PK), thereby being a hindrance to reaching optimal drug concentrations (Food and Drug Administration (FDA) USD of H and HS, 2020).

Administration of aminoglycosides is recommended for newborns at risk of early-onset sepsis, when it is manifested within 72 h after birth. The empirical therapy recommended is gentamicin, alone or in combination with β-lactam antibiotics, due to the high susceptibility of both gram-positive and gram-negative pathogens (National institute for health and care excellence (NICE), 2021). Gentamicin exhibits a concentration-dependent bactericidal effect and requires high peak concentrations (C_max_) to exhibit post-antibiotic effect at drug levels below the minimum inhibitory concentration (MIC). Moreover, high trough concentrations (C_min_) are associated with potential toxicity. Therefore, interactions between pharmacokinetic and pharmacodynamic (PK/PD) parameters should be taken into account for evaluating clinical therapeutic outcomes. PK/PD indexes such as the ratio of C_max_/MIC, the area under the drug concentration–time curve to the MIC (AUC_24_/MIC) or the percentage of time of the dosage interval the drug concentration remains above the MIC (T_>MIC_), are considered the best descriptors of clinical efficacy of antibiotics. For aminoglycosides, the most suitable indices are the ratio C_max_/MIC and the T_>MIC_ (Wicha et al., 2021). In fact, treatment selection is diverging from standard dosing concepts toward the used of PK/PD indexes of each antibiotic for optimal dosing regimen selection, in order to avoid suboptimal drug concentrations which also favour resistance development (Abdul-Aziz et al., 2020). Extended and conventional gentamicin intervals are defined as once-daily or twice-daily in adults, respectively. However, in paediatrics larger dose intervals are suggested due to the lower drug elimination shown in this population (Lanao et al., 2004).

Clinical studies in newborns are rarely performed due to ethical concerns. In fact, the use of *in silico* methods, such as the Physiologically-based pharmacokinetic (PBPK) modelling approach, is recommended for drug development and clinical prescriptions for paediatric populations, in order to reduce the number of child patients in clinical trials (EMA, 2018). PBPK modelling provides the ability to combine maturation and physiological age-related parameters and it has been shown that the predictive performance of a PBPK model is superior to that of empirical/traditional compartmental models (Jones et al., 2006; Jones et al., 2011). Thus, PBPK modelling can be useful for investigating clinical efficacy and safety of current adult treatments in paediatric populations, with this assessment being more accurate than a simple extrapolation based on body weight (Lin et al., 2018). Moreover, model-informed precision dosing (MIPD) is recommended for drugs like gentamicin where adequate exposure is critical, cannot be controlled by easy-to-measure clinical parameters and present large PK variability and a narrow therapeutic index (Wicha et al., 2021). Thus, PBPK models could also be helpful for dosage regimen decision-making in this population.

The aims of this work were to develop a minimal PBPK model of gentamicin in neonates to evaluate potential efficacy and toxicity of the current conventional and extended interval gentamicin dosage regimens in neonates, in order to support MIPD strategies based on PK/PD criteria. This work differs from the previous ones (Abduljalil et al., 2020; Neeli et al., 2021) in several aspects, both in relation to the population study (preterm and term neonates), the methodology and novel PBPK modelling and simulation (M&S) software used (PhysPK), and the used of PK/PD indexes in order to be more comprehensive for clinicians.

## 2 Materials and methods

### 2.1 Physiologically based pharmacokinetic model development

#### 2.1.1 Model development

The PBPK model development was based on gentamicin properties such as quickly extracellular water distribution, minimal binding to plasma proteins and low intracellular penetration. Moreover, taking into account that the kidney is a pivotal organ related to gentamicin elimination, a minimal PBPK model was adopted including two flow-limited compartments: kidney and rest of tissues. Both tissues were connected by the circulating blood system and heart chamber, and defined by tissue volume, blood flow and partition coefficient. Since gentamicin undergoes glomerular filtration and tubular reabsorption, this last process has been included in the mathematical description of the kidney compartment by a 21% correction of the initial drug filtered by the kidney (Contrepois et al., 1985).

#### 2.1.2 Model equations

The minimal PBPK model was defined by simulation component relationships and mathematical equations of the mechanism. The following sections define the mathematical equations for each component.

In the PhysPK model, the heart is not modelled as a tissue but as the source of cardiac output (CO). That is, it does not address ADME processes but only the cardiac flow pumping through a well-stirred chamber, governed by Eq. 1:

$$V_{h}*\frac{{dC}_{h}}{dt}=\left(Q_{h}*C_{b}\right)-\left(Q_{h}*C_{h}\right)$$ (1)

where V_h_ is the heart chamber volume, C_h_ is the drug concentration in the heart, C_b_ is the blood serum drug concentration, and Q_h_ is the heart flow rate (for which CO value has been used). The equation describes the mechanistic effect of the blood mix with the inertia (C_h_ tends to C_b_ with a delay) related to the volume V_h_.

Kidney tissue was considered as a flow-limited tissue and gentamicin tissue concentration is derived from blood concentration according to flow limited tissue (FLT) assumption, which is provided by Eq. 2:

$$V_{k}*\frac{{dC}_{k}}{dt}=\left(Q_{k}*C_{b}\right)-\left(Q_{k}*\frac{C_{k}}{P_{k}}\right)-\left(Q_{e}*\frac{C_{k}}{P_{k}}\right)$$ (2)

where V_k_ is the kidney volume, C_k_ is the drug concentration in the kidney, C_b_ is the blood serum drug concentration, Q_k_ is the kidney flow rate, P_k_ is the partition coefficient of this tissue and Q_e_ is the elimination flow rate.

Linear behaviour was assumed in order to model kidney elimination of the drug through the kidney. Tubular reabsorption has been considered in GFR adjustment while tubular secretion is negligible in the case of gentamicin, so GFR x TBW is expressed in mL/min, considering that 21% of the drug has tubular reabsorption (Eq. 3):

$$Q_{e}=TBW*GFR*0.79$$ (3)

where Q_e_ is the elimination flow rate, GFR is the glomerular filtration rate parameter expressed in mL/min/kg and TBW is the total body weight.

Rest tissue includes the remaining body tissues not explicitly considered. It is defined as a flow-limited tissue and gentamicin tissue concentration is provided by Eq. 4:

$$V_{r}*\frac{{dC}_{r}}{dt}=\left(Q_{r}*C_{b}\right)-\left(Q_{r}*\frac{C_{r}}{P_{r}}\right)$$ (4)

where V_r_ is the volume of the rest tissues (all except heart and kidney), C_b_ is the blood serum drug concentration, C_r_ is the drug concentration in this compartment, Q_r_ is the blood flow rate for this compartment and P_r_ is the partition coefficient of this compartment.

Serum is defined by the blood system, however, gentamicin binding to plasmatic proteins or cells has not been considered relevant since it was less than 10% (Burton, 2006). Thus, the cells compartment has not been considered in the model development and serum concentration is governed by Eq. 5:

$$Vd*\frac{{dC}_{b}}{dt}=G*P\left(t\right)+\left(Q_{h}*C_{h}\right)+\sum Q_{i}\frac{C_{i}}{P_{i}}-\left(Q_{h}{*C}_{b}\right)$$ (5)

where Vd is the volume distribution, C_b_ is the blood serum drug concentration, C_h_ is the drug concentration in the heart, G is the dose of gentamicin administered, P(t) is a unitary pulse waveform with period T (equivalent to interval administration) and pulse width of 30 min and C_i_, Q_i_, P_i_ are the drug concentration, flow rate and partition coefficient for each compartmental tissue (kidney and rest), respectively.

#### 2.1.3 Model parameters

General physiological parameters such as organ volumes, blood flow rates and partition coefficient were already implemented in the software (Table 1). Partition coefficient of gentamicin in kidney was considered as an approach from previous studies (Thibault et al., 1994). Physiological parameters selected based on previous knowledge in preterm and term neonates, and used during the model-building process were: total body weight (TBW), cardiac output (CO), glomerular filtration rate (GFR), gentamicin volume of distribution (Vd) and kidney flow rate (Qk) (Table 2) (Izquierdo et al., 1992; Jackson et al., 1999; Hayton, 2000; Ali et al., 2012; Encinas et al., 2013; Sulemanji and Vakili, 2013). Monte Carlo simulation methodology was used to take into account the variability of these meaningful physiological parameters. It was assumed that they follow a log-normal distribution, and have coefficients of variation (CV) of 20% for TBW and Qk, 24% for CO and 40% for GFR (Bouillon-Pichault et al., 2011; Wilhelm-Bals et al., 2020).

**TABLE 1:** General physiological parameters values implemented in the gentamicin minimal PBPK model.

	Kidney	Rest of tissues
Organ volumes (ml)	0.03	0.87
Blood flow rates (ml/min/kg)	9.35	217
Partition coefficient	10.0	1.00

**TABLE 2:** Physiological parameters values implemented in the gentamicin minimal PBPK model.

Physiological parameter	Preterm population [mean ± SD]	Term population [mean ± SD]
Total body weight (kg)	1.73 ± 0.35	3.56 ± 0.72
Cardiac output (ml/min/kg)	172 ± 50.6	172 ± 50.6
Glomerular filtration rate (ml/min/kg)	1.31 ± 0.53	1.72 ± 0.70
Gentamicin volume of distribution (L/kg)	0.52 ± 0.16	0.46 ± 0.14

SD, standard deviation.

#### 2.1.4 Model validation

Gentamicin minimal PBPK model-based predictions of concentrations by Monte Carlo Simulation were compared to retrospective gentamicin serum concentrations measured in neonates treated at the University Hospital in Salamanca (Spain). Predictions of drug clearance were compared to the clearance values estimated by Maximum a Posteriori (MAP) Bayesian forecasting routinely performed in the TDM (defined as CL observed). For the prediction performance of the model, the prediction error (PE; Eq. 6) and mean prediction error (MPE; Eq.7) were calculated.

$$PE\;\left(\%\right)=\frac{PRED-OBS}{OBS}\times 100$$ (6)

$$MPE=\frac{1}{n}\sum PE$$ (7)

The overall predictability of this model was evaluated in terms of bias and precision using the conventional metrics of average-fold error (AFE; Eq. 8) and absolute average-fold error (AAFE; Eq. 9), respectively.

$$AFE=10^{\frac{1}{n}\sum \log\left(\frac{PRED}{OBS}\right)}$$ (8)

$$AAFE=10^{\frac{1}{n}\sum |\log\left(\frac{PRED}{OBS}\right)|}$$ (9)

If the model predictions reached the criteria of the AFE and AAFE between 0.5 and 2-fold, its predictive performance would be considered to be satisfactory (Puttrevu et al., 2020; Corral Alaejos et al., 2022).

Visual predicted check (VPC) was carried out to evaluate PBPK model performance based on simulations. Observed concentrations were dose-normalized and expressed as the standard dose considered in the Monte Carlo simulations for each group (6 mg/kg q48h for preterm neonates and 6 mg/kg q36h for term neonates). A total of 1,000 virtual patients of each subpopulation were considered to calculate prediction intervals (PI) of 90% and 50%. If observed concentrations were distributed within the 90% PI, the model prediction capability was deemed to be adequate (Rubinstein and Kroese, 2016). Finally, the VPC plots were generated in R version 4.0.2 software from the output of the simulations performed in PhysPK.

### 2.2 Software

Gentamicin minimal PBPK model was developed using the commercially available PhysPK v.2.4.1 platform as part of EcosimPro 6.2.0^®^ (www.physpk.com). This software is based on first-principles modelling of complex systems with continuous and discrete time equations; which use the Multi-Object-Oriented Modelling (MOOM) paradigm. EcosimPro language is designed to model systems formulated through differential-algebraic equations (DAE) and discrete events, by means of a non-algorithmic code (acausal simulation language). Any EcosimPro model is converted to algorithmic code (C++) through the EcosimPro platform previously being executed. PhysPK is a PBPK M&S software built by means of the EcosimPro language. Simulation component parameters and mechanism variables are defined in the International System of Units (SI), although the user parameter values can be defined with other units and converted to SI with internal functions of PhysPK (Martí et al., 2014).

The gentamicin PBPK model has been created through two categories of mathematical equations. The first one is defined by the physical processes that occur inside each simulation component. These processes are liberation, absorption, distribution, metabolism and excretion (LADME) and other mechanisms for the cardiac output source and drug administrator. Mathematical equations in a simulation component describe these processes through differential - algebraic equations (DAE) to give mass conservation, metabolism, absorption, distribution, excretion, and others (inertial pumping or drug rate infusion) for each chemical compound inside all the spatial regions that pertain to the component (Roa and Prado, 2006). The second category refers to the equations that describe the relationships among the simulation components in the PBPK model. The last equations define the blood convection through the vascular system in our PBPK model. They are generated by PhysPK according to the blood connections. Once a PhysPK model is created, it may be used to predict the evolution of the system starting from the initial conditions for a particular context through a simulation experiment.

A second important issue is that the model’s equations are represented by means of a non-algorithmic mathematical formulation. That is, the model is defined in a balanced and complete way (number of variables to solve are equal to number of equations), but it must be previously converted to a flat and algorithmic code in order to be executed in the simulation experiment. The algorithmic code defines the order in which variables are computed and which equations are used to solve them. This methodology for DAE systems is more fully described in the Wiley Encyclopedia of Biomedical Engineering (Roa and Prado, 2006).

EcosimPro also supports Montecarlo simulations, generating a range of possible outcomes considering the *a priori* likelihood of different variables or scenarios. Moreover, the successive productions of random numbers will also be the same using a seed. The random numbers generated, for this PBPK model, have log-normal probability density function.

### 2.3 Clinical data

The study was carried out using data from 47 newborn patients admitted between 1999 and 2003 in the Neonatology Unit of the University Hospital in Salamanca (Spain). These data were obtained as part of routine therapeutic drug monitoring (TDM) of aminoglycoside therapy in paediatric patients suspected of suffering from infection due to Gram-negative microorganisms, regardless of whether this had been confirmed in the antibiogram. This data has also been used by our group in a previous publication (Lanao et al., 2004). Patients selected are characterized to be less than one-week of PNA and with GA between 32 and 39 weeks. These patients were subsequently divided into two subgroups due to their different physiological maturation: preterm neonates with GA between 32 and 37 weeks, and term neonates with GA over 37 weeks. Their concentration-time data were used for the model validation.

#### 2.3.1 Gentamicin dosing and sampling schedule

Gentamicin was administered in the form of an intravenous (IV) infusion during 0.5 h, with an initial dose of 6 mg/kg and an administration interval of 36 h or 48 h. Blood serum samples were collected at 2 and 24 h from the start of drug infusion (Lanao et al., 2004).

Serum gentamicin levels were measured by fluorescence polarization immunoassay AXYM (Abbott Laboratories, Chicago, IL, United States) The method was successfully verified for the calibration range of 0.3–10 mg/L. Additional details of the analytical method are provided in a previous publication (Lanao et al., 2004).

### 2.4 Clearance estimation

In order to check the functionality of the software and the suitability of GFR values selected from literature for the PBPK model developed, this parameter was also estimated using the PhysPK parameter estimation module.

This module allows for the estimation of population parameters of non-linear physiological models. The iterative two-stage (ITS) method was applied to estimate an initial condition of population parameters for each individual subject, without taking into account the population knowledge (Davidian, 2010). After that, the population parameters were estimated using a first-order conditional estimation (FOCE) method, starting with values obtained in ITS.

### 2.5 Gentamicin standard dosage evaluation based on PK/PD criteria

Based on PK/PD criteria, conventional and extended interval dosage regimens were evaluated for each subpopulation. For preterm neonates the dosage regimens evaluated were 4 mg/kg/day (q24h) as a conventional regimen, and 6 mg/kg/day (q48h) as an extended interval regimen; while for term neonates the dosages selected were 4 mg/kg/day (q24h) and 6 mg/kg/day (q36), respectively (Lanao et al., 2004). The PTA of different PK/PD indexes were selected for treatment efficacy and toxicity criteria evaluation.

C_max_/MIC ratio over 8–10 folds, keeping C_max_ lower than 25 mg/L, are necessary to achieve post-antibiotic effect warranting an adequate safety profile related to the maximum drug exposure. Moreover, C_min_ lower than 2 mg/L has been suggested to minimize the potential toxic effects of gentamicin, because it is associated with lower accumulation in both the renal tubule and inner ear (Vučićević et al., 2014). Therefore, the target PK/PD criteria for toxicity were established at Cmin ≥2 mg/L and Cmax ≥25 mg/L. Regarding efficacy criteria, C_max_/MIC ≥8 and T_>MIC_ (expressed as percentage of the dosing interval) equal to or higher than 60% were selected for both dosage regimens (Zazo et al., 2013). Based on the fact that gentamicin is the treatment recommend against pathogens with MIC values between 0.5 and 2 (O’Connor et al., 2021), MIC value of 1 mg/L was selected for PK/PD criteria calculations. For aminoglycosides, the treatment response was defined as effective when the PTA ≥90% for efficacy PK/PD criteria, and for safety when PTA ≤10% for toxicity criterion (He et al., 2022).

## 3 Results

**FIGURE 1:**
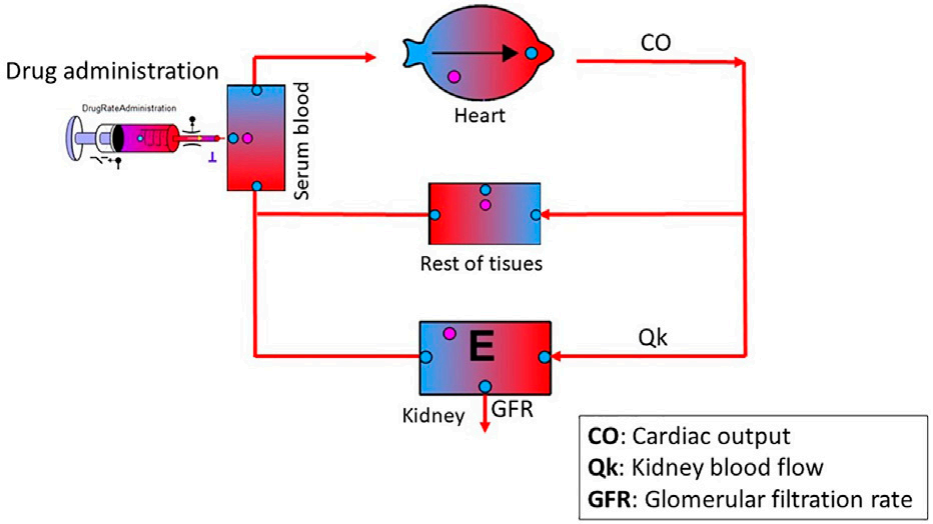
Schematic diagram of the minimal PBPK model developed in PhysPK. Black solid circles are Boolean components for activation/deactivation of processes (i.e., dose administration); pink and blue solid circles, are recording components of the system (i.e., drug concentration in a specific region of the system such as plasma, tissue, etc.,). Organ with an E inside means that it has elimination. The purple T next to the syringe means that the administration has a rate and it can be multiple.

The schematic diagram of the minimal PBPK model developed in the interface of PhysPK is shown in Figure 1. It included the two tissues modelled, kidney and rest of tissues, and the heart as the source of CO. The relationships among simulation components are given by the multilevel modelling schematic.

A total of 91 serum samples from 47 subjects, were used for the minimal PBPK model development. Demographic characteristics of patients evaluated are shown in Table 3.

**TABLE 3:** Demographic baseline characteristics of neonates’ patients evaluated.

Parameter	Preterm (GA ≤37 weeks) [mean ± SD (min-max)]	Term (GA >37 weeks) [mean ± SD (min-max)]
Number of patients	31	16
Gestational age (GA) (weeks)	33.7±1.56 (32-37)	38.9± 0.25 (38-39)
Postnatal age (PNA) (days)	3.03±1.02 (2-7)	2.94±0.85 (2-5)
Total body weight (kg)	1.96 ± 0.41 (1.16–3.00)	3.09 ± 0.23 (2.29–3.62)
Dose (mg/kg)	5.74 ± 0.80 (4.01–6.88)	6.64 ± 0.22 (6.99–6.21)
Serum concentration at 2 h	17.7 ± 4.28 (5.06–24.1)	13.8 ± 3.08 (8.17–20.1)
Since drug administration (mg/L)		
Serum concentration at 24 h	2.92 ± 0.79 (1.84–5.50)	1.41 ± 0.41 (0.86–2.32)
Since drug administration (mg/L)		

SD, standard deviation.

**FIGURE 2:**
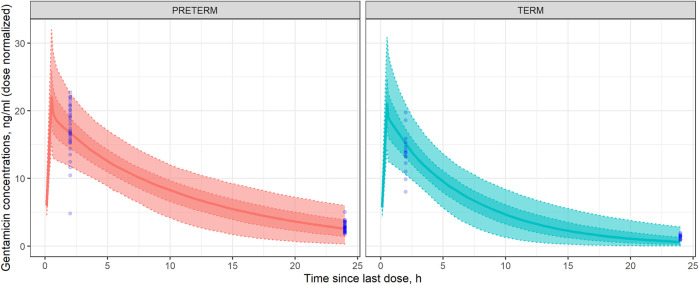
Visual Predicted Check (VPC) in preterm (left) and term (right) neonates. Dotted lines represent, from the bottom to the upper panel, the 10th, 25th, 75th, and 90th percentiles of the gentamicin concentrations simulated vs. time. Shaded areas represent, from outside to inside, the 90% and 50% prediction intervals. Solid lines represent the median gentamicin simulated concentration-time profile. Open blue circles represent the observed gentamicin concentrations dose-normalized.

Most of gentamicin observed concentrations (80.6% and 100% for the C_2h_ and C_24h,_ respectively for preterm neonates, and 87.5% and 100% for the C_2h_ and C_24h,_ respectively for term neonates) were within the 90% PI. Therefore, it can be assured that the minimal PBPK model developed for gentamicin in neonates adequately describes the drug concentration-time profile observed, as well as its PK variability, in both the preterm and term neonate populations studied (Figure 2).

Table 4 shows the mean of observed and predicted concentration and clearance values, as well as the bias and precision evaluation for the validation of the model-predictive performance. Both, AFE and AAFE values for all the predictions were between 0.5 and 2. In fact, all AFE values are nearly 1 which is indicative of a lack of significant bias associated with model predictions.

**TABLE 4:** Mean values and precision and bias errors of gentamicin minimal PBPK model developed.

	Preterm			Term		
	C 2 h (mg/L)	C 24 h (mg/L)	CL (L/h)	C 2 h (mg/L)	C 24 h (mg/L)	CL (L/h)
Mean value observed	17.7	2.92	0.09	13.8	1.41	0.19
Mean value predicted	17.4	2.62	0.11	15.4	0.89	0.23
Mpe (%)	8.08	−5.04	26.5	16.3	−28.8	27.7
Afe	1.02	0.92	1.24	1.14	0.68	1.26
Aafe	1.22	1.23	1.26	1.20	1.47	1.29

C, concentration; CL, clearance; MPE, mean prediction error; AFE, average-fold error; AAFE, absolute average-fold error.

The GFR values obtained by optimization with the PhysPk software were (mean ± SD) 1.176 ± 0.037 ml/min/kg and 1.324 ± 0.054 ml/min/kg for preterm and term neonates, respectively.

**FIGURE 3:**
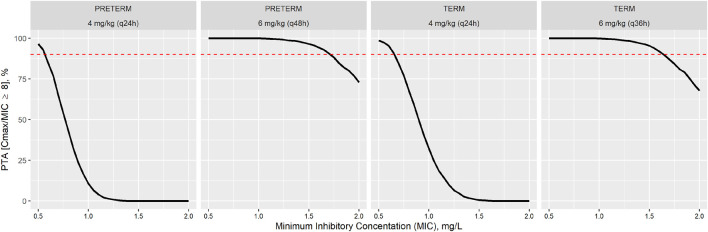
Probability of target attainment (PTA) to reach the C_max_/MIC ≥8 ratio for each gentamicin dosage regimen: conventional (4 mg/kg q24h) and extended interval regimen (6 mg/kg q48h and 6 mg/kg q36h for preterm and term, respectively).

Figure 3 shows that the PTA to reach a C_max_/MIC was adequate with the extended interval regimen (6 mg/kg q48h and 6 mg/kg q36h for preterm and term, respectively) while PTA reached with the conventional regimen (4 mg/kg q24h) was insufficient to achieve proper treatment efficacy, for any MIC value considered (0–2 mg/L). In fact, the extended interval regimens can be assumed to be effective (PTA≥90%) up to MIC values nearly 2 mg/L (1.7 mg/L for preterm and 1.6 mg/L for term neonates), while conventional regimens can be considered effective until MIC values close to 0.6 mg/L (0.55 mg/L for preterm and 0.65 mg/L for term neonates).

Table 5 show the PTA to reach the efficacy and toxicity criteria (C_max_/MIC ≥8 and T_>MIC_ ≥60% for efficacy and C_min_ ≥2 mg/L and C_max_ ≥25 mg/L for toxicity) for each gentamicin regimen considered in each specific population. According to the PTA of PK/PD indexes studied, both regimens presented PTA <10% for the toxicity criteria.

**TABLE 5:** Probability of target attainment (PTA) for efficacy and toxicity criteria (%) (MIC = 1 mg/L).

PK/PD criterion	Preterm		Term	
	Conventional Reg. (q24h)	Extended interval (q48h)	Conventional Reg. (q24h)	Extended interval (q36h)
Efficacy				
C_max_/MIC ≥8	10.8	99.9	32.3	99.8
T_>MIC_ ≥60%	87.9	70.7	61.2	54.9
Toxicity				
C_min_ ≥2 mg/L	5.0	5.9	0.4	1.8
C_max_ ≥25 mg/L	0.0	6.9	0.0	6.5

C_min_, trough concentrations; C_max_, peak concentrations; MIC, minimum inhibitory concentration; PK/PD, pharmacokinetic-pharmacodynamic; T_>MIC_, percentage of time of the interval the drug concentration remains above the MIC.

## 4 Discussion

M&S techniques in biomedicine have experienced great advances and impact in drug development and evaluation in recent decades. Consequently, specialized M&S software is now necessary in the pharma, clinical and biomedical engineering industries. Neonates are one of the most vulnerable populations and also require more ethical considerations, so studies designed with M&S methodologies have been highlighted as a useful methodology in drug development, especially for dose selection decision-making.

The software used in this work, PhysPK, is a novel PBPK platform which has already been successfully applied in MIPD of paediatric patients (Prado-Velasco et al., 2020). It is a robust and potent M&S tool allowing easy and efficient implementation of simulations, execution (such as parameter estimation) and dosage optimization algorithms based on PK/PD criteria (efficacy and/or toxicity), which supports the implementation of MIDP strategies in the clinical routine. PhysPK provides a highly intuitive graphics environment that facilitates the PBPK model development process based on object-oriented schematics (Rodrigues Matos et al., 2013). It is based on different multi-flexi libraries which let components’ development and reusability (i.e., tissues, membranes, etc.,) increasing model complexity and overcoming the useful of other PBPK tools, like Simcyp, GastroPlus or PK-SIM (Reig-Lopez et al., 2020). The PhysPK modeling approach let also to customized PBPK models, like MATLAB/Berkeley Madonna, but without mathematical causality requirements like others software, as NONMEM or Winnonlin (Prado-Velasco, 2016) (Table 6). Moreover, models and calculations can be encapsulated and encrypted in a standalone application (deck), for example Shiny applications. Then, user can only input any data from external well-known applications like Excel, Matlab, C++, etc., and visualize the outputs in real time.

**TABLE 6:** Comparison of PhysPK and commonly used PBPK software programs.

	PhysPK	Simcyp	GastroPlus	NONMEM	WinNonlin	PK-Sim/MOBI
Company	Empresarios Agrupados Internacional	Certara United States Inc.	Simulations Plus Inc.	ICON plc	Certara United States Inc.	Bayer Technology Services
User friendly software	Yes	Yes	Yes	Yes	Yes	Yes
Model creation and edition	Yes	No	No	Yes	Yes	Yes
Population estimation/validation module	Yes	Yes	Yes	Yes	No^a^	No^a^
Execution out-of-the-box	Yes	No	No	No	No	No

a Estimation/validation module by optimization.

This work is focused on a neonatal population with less than 1 week of life. This is a critical period of life for newborns where the highest infection mortality rate has been observed (Author Anonymous, 2020). Therefore, patients have been classified based on gestational age (GA), in two important subgroups: 1) Preterm neonates with GA ≤37 weeks and 2) Term neonates with GA >37 weeks. The main differences between these two groups are whole blood volume, physiological maturation, and total body water. Moreover, rapid developmental changes of physiological factors in newborns affect drug PK, so different parameters’ values have been used for each subpopulation. Thus, a gentamicin PBPK model was developed and validated in term and preterm neonates. Subsequently, the model was used to evaluate the efficacy and safety, based on PK/PD criteria, of current gentamicin dosages recommended for these population.

Extended interval aminoglycoside regimens are recommended, rather than conventional multiple daily dose regimens, because they allow achievement of higher C_max_ and lower C_min_, improving therapeutic efficacy and safety (Wicha et al., 2021). These reasons, along with pharmacoeconomic justification, are all sufficient grounds to select extended interval dosing as the gold-standard regimens for gentamicin in adults (Abdul-Aziz et al., 2020). In neonate populations, their use has been suggested by various authors, even the British Medical Association, despite this regimen being off-label (Lanao et al., 2004; National institute for health and care excellence (NICE), 2021; Rao et al., 2011; El-Chaar et al., 2016). However, some guidelines still recommend a once-daily dosage regimen for term neonates instead of extended-interval such as in other populations like preterm ones (ANMF - Australasian Neonatal Medicines Formulary, 2022; Comité de Medicamentos de la Asociación Española de Pediatríaá, 2022). In fact, according to Neeli et al. 60% of term neonates in intensive care units treated with a once-daily dosage regimen presented potentially toxic concentrations but only around a quarter of them were changed to an extended-interval dosage regimen (Neeli et al., 2021). In order to be a more comprehensive study that encourages clinicians to extended-interval regimen for neonates, specially term ones, this research has been focused on both kinds of neonates, preterm and term, unlike other gentamicin PBPK models published which focus just on preterm populations (Abduljalil et al., 2020; Idkaidek et al., 2020).

The PBPK model development follows a top-down modelling approach. Gentamicin is a hydrophilic drug (logP = –3.1) that mostly distributes in extracellular fluid, eliminating the need for the full PBPK model [16]. It also has low intracellular penetration so it was assumed that the drug distributes instantly in the whole volume of the tissue from the incoming blood flow and a flow-limited model was selected. The kidney is a pivotal organ related to gentamicin elimination. Indeed, 90% of the drug is renally excreted, and it is also directly related to gentamicin nephrotoxicity (Johnson et al., 2006). Thus, due to their PK and toxicity interest, this tissue was the only one included in the minimal PBPK model.

Renal function matures slowly and depends on GA and PNA, as indicated by diuresis, GFR and renal tubular activity (Vučićević et al., 2014). In order to take into account kidney maturation, the studied population has been divided into two subgroups, preterm and term neonates, with different mean GFR values selected for each subgroup based on the available information in the scientific literature. Moreover, 21% of gentamicin that is renally excreted undergoes tubular reabsorption (Contrepois et al., 1985) and this was also reflected in the minimal PBPK model proposed. This is an important point of difference vs*.* other studies which used adult GFR values (Gastine et al., 2022) or reflected the absence of tubular reabsorption as a limitation (Abduljalil et al., 2020; Neeli et al., 2021).

Based on the critical relevance of the GFR values observed during the model-building stage, this parameter was also optimized using the PhysPK parameter estimation module both in preterm and term neonates. The GFR values obtained by optimization through the PhysPK software (1.176 ± 0.037 ml/min/kg and 1.324 ± 0.054 ml/min/kg for preterm and term, respectively) were well in agreement with those implemented in the PBPK model from the literature [1.21 ml/min/kg and 1.59 ml/min/kg for preterm and term, respectively (Izquierdo et al., 1992; Sulemanji and Vakili, 2013)]. In this way, both the selected values and the functionality of the software has been verified.

In order to evaluate the appropriateness of the model-based predictions, bias and precision metrics (AFE and AAFE) were calculated with results between 0.5 and 2 in all of the cases (Table 4). For concentrations at 2 h after start of drug administration, the AFE value near 1 indicates a lack of bias associated with model predictions. Despite of there are some values under the 10th percentile and an outlier in preterm population. Although, for concentrations at 24 h the MPE results indicate that in the term population, what are lightly underestimated (Table 4). Simulation-based diagnostics (Figure 3) showed an adequate model prediction-capability of the gentamicin minimal PBPK model in term and preterm neonates. The large PK variability of the neonatal population observed in these patients was in agreement with previous studies (Nielsen et al., 2009; Bijleveld et al., 2017). This gentamicin PK variability was also properly captured by the gentamicin minimal PBPK model-based simulations.

PBPK models are useful both for predicting drug concentrations and PK parameters which are difficult to measure properly, such as the drug clearance in neonates. The measurement of creatinine levels in neonates is not precise because of the reflection of maternal creatinine levels and the uncertainty about creatinine renal tubular handling. So, it makes the use of serum creatinine levels unreliable and therefore the use of the Schwarz Bedside equation is not recommended in patients younger than 1 year old (Sulemanji and Vakili, 2013; Go et al., 2018). According to the bias and precision results (Table 4), CL values predicted with this gentamicin minimal PBPK model are suitable.

Because the therapy usually starts empirically, the causative pathogen and then its MIC value are commonly unknown at this clinical stage, so MIC distributions from EUCAST reference laboratories are typically used. The highest MIC breakpoint for gentamicin is 2 mg/L. In such case that the pathogen isolated presents a higher value, administration of a concomitant antibiotic or a change to another treatment would be advisable (Burton, 2006). Therefore, no MIC higher than 2 mg/L was considered when evaluating Cmax/MIC efficacy criterion (Figure 3) and a reference MIC of 1 mg/L has been selected for PTA calculation as the most likely within the standard MIC observed in the clinic for pathogens treated with gentamicin.

The conventional dosing regimen recommended by the AEP-SEIP has been compared with the extended interval dosage regimen recommended by the University Hospital in Salamanca. Figure 3 showed that both gentamicin dosage regimens appear to be potentially effective and safe, according to the PK/PD indexes considered. Peak serum concentrations of gentamicin with the conventional regimen (4 mg/kg q24h for both populations) were not sufficiently high enough to achieve C_max_/MIC ratio over 8 necessary to achieve post-antibiotic effect, in most cases (Figure 3). Moreover, it could bring on bacterial resistance development despite eliciting a therapeutic response. In contrast, extended interval gentamicin dosing regimens (6 mg/kg q48h and 6 mg/kg q36h for preterm and term neonates, respectively) showed adequate efficacy (PTA higher than 90%) with acceptable safety (PTA for toxicity criteria ≤7% in all cases) through Monte Carlo simulation evaluations (Table 5). Indeed, extended interval regimens reached the main efficacy criteria (C_max_/MIC ≥8) up to MIC values of 1.55 mg/L and 1.65 mg/L for preterm and term neonates, respectively (Figure 3). These results are in line with the current clinical decisions of combining gentamicin with an alternative antibiotic or even changing the drug selected to treat a Gram-negative infection caused by pathogens with MIC higher than 2 mg/L (O’Connor et al., 2021). However, the conventional dosing regimen only reaches C_max_/MIC ≥8 against pathogens with MICs lower than 0.5 mg/L (Figure 3).

Findings in our study are consistent with other published studies like Gastine et al. (2022) whose results show that the conventional regimen would be insufficient to cover most common Gram-negative Enterobacterales (MIC ≤2 mg/L) responsible for many neonatal sepsis, with this regimen being efficacious in only less than one-quarter of neonates treated.

Apart from the important usefulness and potential applications of the gentamicin minimal PBPK model presented in this work, some limitations must be acknowledged such as: 1) The model has been validate with a small sample size, just 2 concentration-time points and retrospective data which might limit precise assessments and additional evaluations of the model; 2) No correlations between the physiological variables simulated were considered in the Monte Carlo simulations (Franchetti and Nolin, 2020). However, the impact of these correlations is not expected to be relevant as most of the parameters were expressed per kg of TBW and specific values observed in the literature were considered for preterm and term neonates.

Finally, gentamicin treatment is limited mainly by its potential toxicity. Thus, this minimal PBPK model will serve as a starting point for future investigations regarding the gentamicin concentrations reached in the kidneys. After a refinement of the minimal PBPK model presented, it will be able to evaluate the potential toxicity of gentamicin dosages proposed in neonates and optimize these dosages based on PK/PD criteria. Moreover, the model presented can be applied to different aminoglycosides in similar populations after model refinement. Similarly, additional information could be valuable for extending the PBPK model applications such as physiological changes in intensive care neonate patients (i.e., fluid shift due to capillary leak and renal dysfunction), kidney and ear accumulation (related to the most-likely drug side effects), or bacteria growth models (considering potential drug resistance) among others.

## 5 Conclusion

In summary, the gentamicin minimal PBPK model developed in the PhysPK platform adequately described preterm and term neonates’ gentamicin PK behaviour. According to the PK/PD criteria, extended interval dosage regimens reached the efficacy criterion with a reduced probability of presenting toxicity even against pathogens with a minimum inhibitory concentration near 2 mg/L. The minimal gentamicin PBPK model presented in this work has shown an adequate model predictive performance with acceptable precision and lack of significant bias. Thus, it could be useful for MIPD strategies in neonates based on PK/PD criteria.

## Data Availability

The original contributions presented in the study are included in the article/Supplementary Material, further inquiries can be directed to the corresponding authors.

## References

[B1] Abdul-Aziz M. H. Alffenaar J. W. C. Bassetti M. Bracht H. Dimopoulos G. Marriott D. (2020). Antimicrobial therapeutic drug monitoring in critically ill adult patients: A position paper. Intensive Care Med. 46 (6), 1127–1153. 10.1007/s00134-020-06050-1 32383061 PMC7223855

[B2] Abduljalil K. Pan X. Pansari A. Jamei M. Johnson T. N. (2020). Preterm physiologically based pharmacokinetic model. Part II: Applications of the model to predict drug pharmacokinetics in the preterm population. Clin. Pharmacokinet. 59 (4), 501–518. 10.1007/s40262-019-00827-4 31587145

[B3] Ali A. S. Farouq M. F. Al-Faify K. A. (2012). Pharmacokinetic approach for optimizing gentamicin use in neonates during the first week of life. Indian J. Pharmacol. 44 (1), 36–40. 10.4103/0253-7613.91864 22345867 PMC3271536

[B4] ANMF - Australasian Neonatal Medicines Formulary (2022). Anmf - australasian neonatal Medicines formulary. [Internet][citado 17 de marzo de 2022]. Disponible en: Available at: https://www.anmfonline.org/ (Accessed March 17, 2022).

[B5] Comité de Medicamentos de la Asociación Española de Pediatríaá, 2022, Pediamécum. Edición 2015 | Asociación Española de Pediatría [Internet]. [citado 7 de febrero de 2022]. Disponible en: Available at: https://www.aeped.es/comite-medicamentos/pediamecum/gentamicina (Accessed February 7, 2022).

[B6] Author Anonymous (2020).Newborns: Improving survival and well-being. Geneva, Switzerland: World Hhealth Organization. [internet]. [citado 3 de diciembre de 2021]. Disponible en:Available at: https://www.who.int/news-room/fact-sheets/detail/newborns-reducing-mortality (Accessed September 19, 2020).

[B7] Bijleveld Y. A. van den Heuvel M. E. Hodiamont C. J. Mathôt R. A. A. de Haan T. R. (2017). Population pharmacokinetics and dosing considerations for gentamicin in newborns with suspected or proven sepsis caused by gram-negative bacteria. Antimicrob. Agents Chemother. 61 (1), e01304–e01316. 10.1128/AAC.01304-16 27795373 PMC5192127

[B8] Bouillon-Pichault M. Jullien V. Bazzoli C. Pons G. Tod M. (2011). Pharmacokinetic design optimization in children and estimation of maturation parameters: Example of cytochrome P450 3A4. J. Pharmacokinet. Pharmacodyn. 38 (1), 25–40. 10.1007/s10928-010-9173-1 21046208

[B9] Burton M. E. (2006). Applied pharmacokinetics & pharmacodynamics: Principles of therapeutic drug monitoring. 4th ed. Baltimore: Lippincott Williams & Wilkins.

[B10] Chan G. J. Lee A. C. Baqui A. H. Tan J. Black R. E. (2013). Risk of early-onset neonatal infection with maternal infection or colonization: A global systematic review and meta-analysis. PLoS Med. 10 (8), e1001502. 10.1371/journal.pmed.1001502 23976885 PMC3747995

[B11] Contrepois A. Brion N. Garaud J. J. Faurisson F. Delatour F. Levy J. C. (1985). Renal disposition of gentamicin, dibekacin, tobramycin, netilmicin, and amikacin in humans. Antimicrob. Agents Chemother. 27 (4), 520–524. 10.1128/aac.27.4.520 4004192 PMC180088

[B12] Corral Alaejos Á. Zarzuelo Castañeda A. Jiménez Cabrera S. Sánchez-Guijo F. Otero M. J. Pérez-Blanco J. S. (2022). External evaluation of population pharmacokinetic models of imatinib in adults diagnosed with chronic myeloid leukaemia. Br. J. Clin. Pharmacol. 88 (4), 1913–1924. 10.1111/bcp.15122 34705297

[B13] Davidian M. (2010). “Nonlinear mixed effects models” in International Encyclopedia of Statistical Science.Editor Lovric M. (New York: Springer).

[B14] El-Chaar G. M. Supaswud-Franks T. Venugopalan L. Kohn N. Castro-Alcaraz S. (2016). Extended-interval gentamicin administration in neonates: A simplified approach. J. Perinatol. 36 (8), 660–665. 10.1038/jp.2016.37 26986995

[B15] EMA (2018).Extrapolation of efficacy and safety in paediatric medicine development. Amsterdam, Netherlands: European Medicines Agency. Internet[citado 10 de febrero de 2022]. Disponible en: Available at: https://www.ema.europa.eu/en/extrapolation-efficacy-safety-paediatric-medicine-development (Accessed 10 30, 2018).

[B16] Encinas E. Calvo R. Lukas J. C. Vozmediano V. Rodriguez M. Suarez E. (2013). A predictive pharmacokinetic/pharmacodynamic model of fentanyl for analgesia/sedation in neonates based on a semi-physiologic approach. Paediatr. Drugs 15 (3), 247–257. 10.1007/s40272-013-0029-1 23657896

[B17] Food and Drug Administration (FDA) USD of H and HS (2020). General clinical Pharmacology considerations for neonatal studies for drugs and biological products guidance for industry. [Internet]. U.S. Food and Drug Administration. FDA[citado 12 de enero de 2022]. Disponible en: Available at: https://www.fda.gov/regulatory-information/search-fda-guidance-documents/general-clinical-pharmacology-considerations-neonatal-studies-drugs-and-biological-products-guidance (Accessed July, 2022).

[B18] Franchetti Y. Nolin T. D. (2020). Dose optimization in kidney disease: Opportunities for PBPK modeling and simulation. J. Clin. Pharmacol. 60, S36–S51. 10.1002/jcph.1741 33205428

[B19] Gastine S. Obiero C. Kane Z. Williams P. Readman J. Murunga S. (2022). Simultaneous pharmacokinetic/pharmacodynamic (PKPD) assessment of ampicillin and gentamicin in the treatment of neonatal sepsis. J. Antimicrob. Chemother. 77 (2), 448–456. 10.1093/jac/dkab413 35107141 PMC8809196

[B20] Go H. Momoi N. Kashiwabara N. Haneda K. Chishiki M. Imamura T. (2018). Neonatal and maternal serum creatinine levels during the early postnatal period in preterm and term infants. PLoS One 13 (5), e0196721. 10.1371/journal.pone.0196721 29795567 PMC5967735

[B21] Hayton W. L. (2000). Maturation and growth of renal function: Dosing renally cleared drugs in children. AAPS PharmSci 2 (1), E3. 10.1208/ps020103 11741219 PMC2750998

[B22] He S. Cheng Z. Xie F. (2022). Population pharmacokinetics and dosing optimization of gentamicin in critically ill patients undergoing continuous renal replacement therapy. Drug Des. devel. Ther. 16, 13–22. 10.2147/DDDT.S343385 PMC8747548 35023902

[B23] Idkaidek N. Hamadi S. Bani-Domi R. Al-Adham I. Alsmadi M. Awaysheh F. (2020). Saliva versus plasma therapeutic drug monitoring of gentamicin in Jordanian preterm infants. Development of a physiologically-based pharmacokinetic (PBPK) model and validation of class II drugs of salivary excretion classification system. Drug Res. 70 (10), 455–462. 10.1055/a-1233-3582 32877949

[B24] Izquierdo M. Lanao J. M. Cervero L. Jimenez N. V. Domínguez-Gil A. Dominguez-Gil A. (1992). Population pharmacokinetics of gentamicin in premature infants. Ther. Drug Monit. 14 (3), 177–183. 10.1097/00007691-199206000-00001 1412601

[B25] Jackson M. Abdel‐Rahman S. Kearns G. (1999). Pharmacology of antibiotics in the neonate. Seminars Pediatr. Infect. Dis. 10, 91–96. 10.1016/S1045-1870(99)80039-9

[B26] Johnson T. N. Rostami-Hodjegan A. Tucker G. T. (2006). Prediction of the clearance of eleven drugs and associated variability in neonates, infants and children. Clin. Pharmacokinet. 45 (9), 931–956. 10.2165/00003088-200645090-00005 16928154

[B27] Jones H. M. Gardner I. B. Collard W. T. Stanley P. J. Oxley P. Hosea N. A. (2011). Simulation of human intravenous and oral pharmacokinetics of 21 diverse compounds using physiologically based pharmacokinetic modelling. Clin. Pharmacokinet. 50 (5), 331–347. 10.2165/11539680-000000000-00000 21456633

[B28] Jones H. M. Parrott N. Jorga K. Lavé T. (2006). A novel strategy for physiologically based predictions of human pharmacokinetics. Clin. Pharmacokinet. 45 (5), 511–542. 10.2165/00003088-200645050-00006 16640456

[B29] Lanao J. M. Calvo M. V. Mesa J. A. Martín-Suárez A. Carbajosa M. T. Miguelez F. (2004). Pharmacokinetic basis for the use of extended interval dosage regimens of gentamicin in neonates. J. Antimicrob. Chemother. 54 (1), 193–198. 10.1093/jac/dkh261 15150171

[B30] Lin W. Yan J. H. Heimbach T. He H. (2018). Pediatric physiologically based pharmacokinetic model development: Current status and challenges. Curr. Pharmacol. Rep. 4 (6), 491–501. 10.1007/s40495-018-0162-1

[B31] Martí R. Rodríguez T. Pitarch J. L. Sarabia D. de Prada C. Dynamic Optimization by automatic differentiation using EcosimPro y Casadi. 2014;12. https://www.ecosimpro.com/wp-content/uploads/2015/02/CEA_2014_dynamic_optimization_EcosimPro_CASADI.pdf

[B32] National institute for health and care excellence (NICE) (2021). “Neonatal infection: Antibiotics for prevention and treatment,” in Guidance NICE (NICE). Overview | Internet[citado 1 de abril de 2022]. Disponible en: Available at: https://www.nice.org.uk/guidance/ng195 (Accessed April 20, 2021). 34133110

[B33] Neeli H. Hanna N. Abduljalil K. Cusumano J. Taft D. R. (2021). Application of physiologically based pharmacokinetic-pharmacodynamic modeling in preterm neonates to guide gentamicin dosing decisions and predict antibacterial effect. J. Clin. Pharmacol. 61 (10), 1356–1365. 10.1002/jcph.1890 33945155

[B34] Nielsen E. I. Sandström M. Honoré P. H. Ewald U. Friberg L. E. (2009). Developmental pharmacokinetics of gentamicin in preterm and term neonates: Population modelling of a prospective study. Clin. Pharmacokinet. 48 (4), 253–263. 10.2165/00003088-200948040-00003 19492870

[B35] O’Connor K. Davies M. W. Koorts P. Cartwright D. W. Whitfield K. (2021). Gentamicin dosing in neonates with normal renal function: Trough and peak levels. Eur. J. Drug Metab. Pharmacokinet. 46 (5), 677–684. 10.1007/s13318-021-00708-x 34370216

[B36] Prado-Velasco M. Borobia A. Carcas-Sansuan A. (2020). Predictive engines based on pharmacokinetics modelling for tacrolimus personalized dosage in paediatric renal transplant patients. Sci. Rep. 10 (1), 7542. 10.1038/s41598-020-64189-9 32371893 PMC7200804

[B37] Prado-Velasco M. (2016). Bridging the gap between open and specialized modelling tools in PBPK/PK/PD with physPK/EcosimPro modelling system: PBPK model of methotrexate and 6-mercaptopurine in humans with focus in reusability and multilevel modelling features. in Proceedings of the Conference: Annual Meeting of the Population Approach Group in Europe (PAGE) 2016, June 2016, Lisboa, Portugal, Internet[citado 17 de marzo de 2022]. Disponible en: Available at: https://www.page-meeting.org/?abstract=5769 .

[B38] Puttrevu S. K. Arora S. Polak S. Patel N. K. (2020). Physiologically based pharmacokinetic modeling of transdermal selegiline and its metabolites for the evaluation of disposition differences between healthy and special populations. Pharmaceutics 12 (10), 942. 10.3390/pharmaceutics12100942 PMC7600566 33008144

[B39] Rao S. C. Srinivasjois R. Hagan R. Ahmed M. (2011). One dose per day compared to multiple doses per day of gentamicin for treatment of suspected or proven sepsis in neonates. Cochrane Database Syst. Rev., CD005091. 10.1002/14651858.CD005091.pub3 27921299 PMC6464017

[B40] Reig-Lopez J. Merino-Sanjuan M. Mangas-Sanjuan V. Prado-Velasco M. (2020). A multilevel object-oriented modelling methodology for physiologically-based pharmacokinetics (PBPK): Evaluation with a semi-mechanistic pharmacokinetic model. Comput. Methods Programs Biomed. 189, 105322. 10.1016/j.cmpb.2020.105322 31954235

[B41] Roa L. Prado M. (2006). “Simulation languages,” in Wiley Encyclopedia of biomedical engineering (New Jersey, United States: John Wiley & Sons). [Internet][citado 16 de marzo de 2022]. Disponible en: Available at: https://onlinelibrary.wiley.com/doi/abs/10.1002/9780471740360.ebs1089 (Accessed April 14, 2006).

[B42] Rodrigues Matos T. Prado-Velasco M. Navarro J. M. Vallez C. (2013). On a reusable and multilevel methodology for modeling and simulation of pharmacokinetic-physiological systems: A preliminary study. Comput. Biol. Med. 43 (10), 1512–1522. 10.1016/j.compbiomed.2013.07.025 24034743

[B43] Rubinstein R. Y. Kroese D. P. (2016). Simulation and the Monte Carlo method. New Jersey, United States: John Wiley & Sons, 432.

[B44] Sulemanji M. Vakili K. (2013). Neonatal renal physiology. Semin. Pediatr. Surg. 22 (4), 195–198. 10.1053/j.sempedsurg.2013.10.008 24331094

[B45] Thibault N. Grenier L. Simard M. Bergeron M. G. Beauchamp D. (1994). Attenuation by daptomycin of gentamicin-induced experimental nephrotoxicity. Antimicrob. Agents Chemother. 38 (5), 1027–1035. 10.1128/aac.38.5.1027 8067733 PMC188145

[B46] Vučićević K. Rakonjac Z. Janković B. Kovačević S. V. Miljković B. Prostran M. (2014). Clinical pharmacokinetics in optimal gentamicin dosing regimen in neonates. Open Med. (Wars). 9 (3), 485–490. 10.2478/s11536-013-0298-7

[B47] Wicha S. G. Märtson A. G. Nielsen E. I. Koch B. C. P. Friberg L. E. Alffenaar J. W. (2021). From therapeutic drug monitoring to model-informed precision dosing for antibiotics. Clin. Pharmacol. Ther. 109 (4), 928–941. 10.1002/cpt.2202 33565627

[B48] Wilhelm-Bals A. Combescure C. Chehade H. Daali Y. Parvex P. (2020). Variables of interest to predict glomerular filtration rate in preterm newborns in the first days of life. Pediatr. Nephrol. 35 (4), 703–712. 10.1007/s00467-019-04257-z 31001662

[B49] Zazo H. Martín-Suárez A. Lanao J. M. (2013). Evaluating amikacin dosage regimens in intensive care unit patients: A pharmacokinetic/pharmacodynamic analysis using Monte Carlo simulation. Int. J. Antimicrob. Agents 42 (2), 155–160. 10.1016/j.ijantimicag.2013.04.021 23756322

